# Differential Effects of Silibinin A on Mitochondrial Function in Neuronal PC12 and HepG2 Liver Cells

**DOI:** 10.1155/2019/1652609

**Published:** 2019-11-23

**Authors:** Carsten Esselun, Bastian Bruns, Stephanie Hagl, Rekha Grewal, Gunter P. Eckert

**Affiliations:** ^1^Institute for Nutritional Sciences, Justus-Liebig-University of Giessen, Giessen, Germany; ^2^Institute of Pharmacology, Goethe-University of Frankfurt am Main, Frankfurt am Main, Germany

## Abstract

The Mediterranean plant *Silybum marianum* L., commonly known as milk thistle, has been used for centuries to treat liver disorders. The flavonolignan silibinin represents a natural antioxidant and the main bioactive ingredient of silymarin (silybin), a standard extract of its seeds. Mitochondrial dysfunction and the associated generation of reactive oxygen/nitrogen species (ROS/RNS) are involved in the development of chronic liver and age-related neurodegenerative diseases. Silibinin A (SIL A) is one of two diastereomers found in silymarin and was used to evaluate the effects of silymarin on mitochondrial parameters including mitochondrial membrane potential and ATP production with and without sodium nitroprusside- (SNP-) induced nitrosative stress, oxidative phosphorylation, and citrate synthase activity in HepG2 and PC12 cells. Both cell lines were influenced by SIL A, but at different concentrations. SIL A significantly weakened nitrosative stress in both cell lines. Low concentrations not only maintained protective properties but also increased basal mitochondrial membrane potential (MMP) and adenosine triphosphate (ATP) levels. However, these effects could not be associated with oxidative phosphorylation. On the other side, high concentrations of SIL A significantly decreased MMP and ATP levels. Although SIL A did not provide a general improvement of the mitochondrial function, our findings show that SIL A protects against SNP-induced nitrosative stress at the level of mitochondria making it potentially beneficial against neurological disorders.

## 1. Introduction

**Figure 1:**
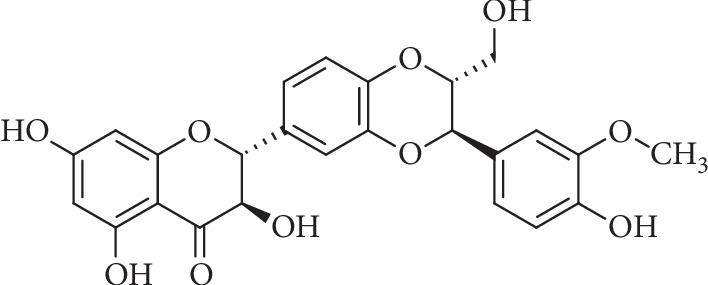
Chemical structure of SIL A (C_25_H_22_O_10_) ((2R,3R)-3,5,7-trihydroxy-2-[(2R,3R)-3-(4-hydroxy-3-methoxyphenyl)-2-(hydroxymethyl)-2,3-dihydro-1,4-benzodioxin-6-yl]-2,3-dihydrochromen-4-one).

The Mediterranean plant *Silybum marianum* L. (Gaertn.; (Compositae)), commonly known as milk thistle, has been used for centuries to treat disorders of the liver. The flavonolignan silibinin (Figure 1) represents the main bioactive ingredient of silymarin (silybin), a standard extract of its seeds [1]. Silibinin A (SIL A), which was used in this study, is one of two diastereomers found in silybin [2]. Mitochondrial dysfunction and the associated generation of reactive oxygen species (ROS) are involved in the development of chronic liver diseases, including nonalcoholic fatty, alcohol-associated, and drug-associated liver diseases, as well as hepatitis B and C [3, 4]. Silybin reduced respiration and adenosine triphosphate (ATP) production but increased mitochondrial size and improved mitochondrial cristae organization in cellular models of steatosis and steatohepatitis [5]. In rats with secondary biliary cirrhosis, silybin was found to exert antioxidant effects and induces mitochondrial biogenesis [6]. Furthermore, SIL A was shown to decrease lipotoxicity by attenuating oxidative stress and NF*κ*B activation in nonalcoholic steatohepatitis (NASH) [7, 8].

Functioning as a scavenger for ROS, SIL A is able to reduce lipid peroxidation, resulting in improved protection against apoptosis [2]. It was also proposed that SIL A shows an effect on the permeability of mitochondrial membranes in liver cells, affecting the integration of cholesterol into the lipid bilayer [2]. Furthermore, it has also been shown that SIL A can regulate the Ca^2+^ influx into mitochondria [9]. HepG2 cells are widely used to study the mechanisms of drug actions, and it has already been reported that SIL A reduced oxidative stress in HepG2 cells [10].

Beside hepatic diseases, mitochondrial dysfunction is also involved in the development of noncommunicable diseases of the brain, including Alzheimer's disease (AD) and Parkinson's disease (PD), as well as amyotrophic lateral sclerosis [11–13]. SIL A prevents dopaminergic neuronal loss in cellular and animal models of PD, and it was suggested that its neuroprotective effect might be mediated by the stabilization of the mitochondrial membrane potential (MMP) [14]. In PC12 cells, silybin and SIL A were found to attenuate oxidative and nitrosative stress [15, 16]. Another study demonstrated the effects of neuroprotective hybrid compounds consisting of SIL A conjugated with phenolic acids on PC12 cell differentiation [17].

PC12 cells originate from the pheochromocytoma of the rat adrenal medulla and are widely established as a neuronal model [18, 19] in differentiated [20, 21] or undifferentiated [22, 23] form.

Recently, it has been reported that SIL A-induced ROS generation protects PC12 cells from sodium nitroprusside- (SNP-) induced nitrosative stress [15]. Nitrosative stress in the form of nitric oxide radicals plays a role in the aging process and in neurodegenerative diseases by contributing to inflammation, neuronal loss, and oxidative stress [24, 25]. SNP is a source widely used to introduce nitrosative stress into cellular models [15, 26].

An improvement of mitochondrial energy metabolism might be a mechanism contributing to the hepatic or neuroprotective action of SIL A, a hypothesis that has not yet been studied in detail. Thus, in this work, we evaluated the effects of SIL A on oxidative phosphorylation, MMP, ATP levels, and citrate synthase activity in PC12 cells and HepG2 cells, both subjected to and without SNP-induced nitrosative stress.

## 2. Material and Methods

### 2.1. Chemicals

All chemicals used for this research were of the highest purity available and were purchased from either Sigma Aldrich, Merck, or VWR. Silibinin A (SIL A) (purity 97%) was ordered from LKT Laboratories. SIL A was solubilized in DMSO, and thus, DMSO (0.1%) was used as a control for all experiments. DMSO had no effect on any of the parameters measured (data not shown). Aqueous solutions were prepared with type-1 ultrapure water.

### 2.2. Cell Lines

Cells used for all experiments were undifferentiated PC12 [27] and HepG2 cells [28], as previously published [29, 30].

PC12 cells were cultivated in 250 mL Greiner flasks with Dulbecco's modified Eagle medium (DMEM) (Gibco, Thermo Scientific) supplemented with 10% (*v*/*v*) fetal bovine serum (FBS), 5% horse serum (HS), and 1% antibiotics (penicillin, streptomycin, and G418). Twice a week, cells were split to maintain cell health and to prevent overgrowth.

HepG2 cells were cultivated in 250 mL Greiner flasks with Dulbecco's modified Eagle medium (DMEM) (Gibco, Thermo Scientific) supplemented with 10% (*v*/*v*) FBS, 5 U/mL penicillin, and 50 *μ*g/mL streptomycin. Twice a week, cells were split to maintain cell health and to prevent overgrowth.

For experiments, cells were harvested from Greiner flasks, counted using a Neubauer chamber, and diluted to yield a cell suspension of 10^6^ cells/mL. Cells were then transferred into 24-well (MMP, 2 × 10^5^ cells/well) or 96-well plates (ATP, 10^5^ cells/well for HepG2 and 2 × 10^5^ cells/well for PC12 cells). Cells were allowed to attach for 48 h in reduced DMEM (2% FBS, 1% HS) before being exposed to SIL A in various concentrations. To assess the effect of SIL A on nitrosative stress, cells were incubated with 0.5 mM (PC12 cells) or 5 mM (HepG2) SNP 1 hour after SIL A exposure. After 24 h, cells were harvested.

### 2.3. Measurement of Mitochondrial Membrane Potential (MMP)

MMP was measured using the fluorescence dye rhodamine-123 (R123). Cells of either cell line were incubated at 37°C and 5% CO_2_ for 15 min with 0.4 *μ*M R123. Cells were centrifuged at 750 × *g* for 5 min before being washed with Hank's Balanced Salt Solution (HBSS) buffer (supplemented with Mg^2+^, Ca^2+^, and HEPES; pH 7.4; 37°C). Cells were suspended in fresh HBSS buffer before being assessed by the measurement of R123 fluorescence. The excitation wavelength was set to 490 nm and the emission wavelength to 535 nm (Victor X3 2030 multilabel counter, Perkin Elmer). The fluorescence was measured four times and normalized to the cell count or displayed relative to the control groups.

### 2.4. Measurement of ATP Concentrations

To assess the ATP concentrations, a bioluminescence kit ViaLight (Lonza), which is based on the production of light via the reaction of ATP with luciferin, was used. The 96-well plate was removed from the incubator and allowed to cool to room temperature for 10 min. Following incubation with lysis buffer for 10 min, cells were incubated for an additional 5 min with the monitoring reagent. The emitted light was assessed with a luminometer (Victor X3 2030 multilabel counter, Perkin Elmer). Since the emitted light is linearly related to the production of ATP, the concentration could be determined by a standard curve. The results were adjusted to the cell count or displayed relative to the control group.

### 2.5. High-Resolution Respirometry

The respiration of mitochondria was measured using an Oxygraph-2k respirometer (Oroboros) as described earlier [19]. For data evaluation, the software DatLab v. 4.3.2.7 was used. The protocol used to assess mitochondrial respiration was created by Gnaiger [31] and includes the addition of several substrates, inhibitors, and uncouplers to a suspension of cells. Respiration is displayed in different stages of the experiment—(1) endogen: the endogenous respiration of cells; (2) Dig: the addition of 8 *μ*M digitonin to disrupt cell membranes and remove naive substrates; (3) CI_(L)_: respiration after the addition of 10 mM glutamate and 2 mM malate to compensate for proton leaks through the membrane; (4) CI_(P)_: coupled complex I respiration after the addition of 2 mM ADP; (5) CI&CII_(P)_: maximal coupled CI and CII respiration after the addition of 10 mM succinate; (6) CI&CII_(L)_: leak respiration of CI and CII after the addition of 2 *μ*g/mL oligomycin; (7) CI&CII_(E)_: maximal uncoupled CI and CII activity to compensate for increased proton transport into the matrix after the stepwise addition of carbonyl cyanide p-trifluoromethoxyphenylhydrazone (FCCP) up to a total concentration of 0.5 *μ*M; (8) CII_(E)_: uncoupled respiration using only CII substrates after CI inhibition via the addition of 0.5 *μ*M rotenone; (9) CIV_(E)_: maximal uncoupled respiration of CIV after the addition of 2.5 *μ*M antimycin A, which inhibits complex III, as well as the addition of the electron-donator 0.5 mM *N,N,N*′*,N*′-tetramethyl-p-phenylenediamine dihydrochloride (TMPD) and 2 mM of the TMPD-regenerating agent ascorbate. The residual oxygen consumption of enzymes not part of the oxidative phosphorylation was measured after the addition of antimycin A and then subtracted from all stages of the experiment. The addition of 12 mM NaN_3_ at the end of the experiment revealed the oxygen consumption due to the autoxidation of TMPD. This, as well as the residual oxygen consumption, was subtracted from CIV_(E)_.

### 2.6. Citrate Synthase Activity

Cell samples from the respirometry measurements were frozen and stored at −80°C for the assessment of the citrate synthase activity. The samples were allowed to thaw while a reaction medium (0.1 mM 5,5′-dithio-bis-(2-nitrobenzoic acid) (DTNB), 0.5 mM oxaloacetate, 50 *μ*M EDTA, 0.31 mM acetyl coenzyme A, 5 mM triethanolamine hydrochloride, and 0.1 M Tris-HCl) was mixed and heated to 30°C for 5 min. Afterwards, a volume of 200 *μ*L of cells was added to the reaction medium, and the citrate synthase activity was determined spectrophotometrically at 412 nm. For statistical analysis, each sample was measured in triplicate.

### 2.7. Pyruvate and Lactate Contents

Frozen cells, which were previously harvested from 250 mL Greiner flasks after 4 days of growth and 1 day of incubation with 50 *μ*M SIL A or control, were thawed to room temperature. Pyruvate and lactate concentrations were assessed using a pyruvate assay kit (MAK071, Sigma Aldrich) and a lactate assay kit (MAK064, Sigma Aldrich) according to the manufacturer's instructions. Absorbance was measured using a CLARIOstar plate reader (BMG Labtech).

### 2.8. Protein Content

Frozen cells were thawed, and protein contents were determined using a Pierce BCA Protein Assay Kit (Thermo Fisher Scientific) according to the manufacturer's instructions. Absorbance was measured using a CLARIOstar plate reader (BMG Labtech).

### 2.9. Statistics

Unless stated otherwise, data are presented as the means ± SEM. Statistical analysis was performed with either Student's *t*-test or one-way ANOVA followed by Tukey's post hoc test performed using GraphPad Prism version 8.0.1 for Windows (GraphPad Software).

## 3. Results

### 3.1. Comparison between Hepatic Cells and Neuronal Cells

**Figure 2:**
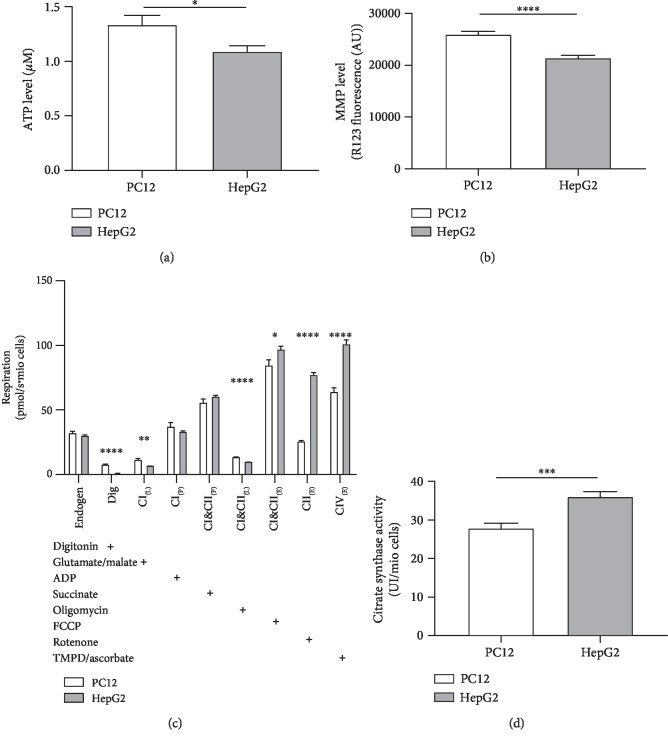
Comparison of mitochondrial function between untreated PC12 and untreated HepG2 cells. (a) Adenosine triphosphate (ATP) level of 10^4^ cells of both cell lines. (b) Mitochondrial membrane potential (MMP) level of 2 × 10^4^ cells of both cell lines. (c) Respiration of 10^6^ cells of both cell lines. Activity of the oxidative phosphorylation (OXPHOS) complexes was assessed via the addition of several substrates, inhibitors, or uncouplers. Which substance was added in which stage of the experiment is indicated by the placement of “+.” Dig = the addition of digitonin; CI_(L)_ = leak respiration of complex I; CI_(P)_ = coupled respiration using only complex I substrates; CI&CII_(P)_ = physiological respiration; CI&CII_(L)_ = leak respiration of complexes I and II; CI&CII_(E)_ = uncoupled respiration using maximum CI&CII activity; CII_(E)_ = uncoupled respiration using complex II substrates only; CIV_(E)_ = uncoupled respiration using only CIV after complex III inhibition and CIV activation using an electron donor. (d) Citrate synthase activity of 10^6^ cells. The procedure for all experiments was the same as that employed for experiments treating cells with SIL A or DMSO, but instead of an effector, the cell medium was used. Data are displayed as the means ± SEM. *N* = 7–18. Statistical significance was tested via Student's *t*-test (^∗∗∗∗^*p* < 0.0001, ^∗∗∗^*p* < 0.001, ^∗∗^*p* < 0.01, and ^∗^*p* < 0.5).

Our data showed that mitochondrial parameters measured in hepatic HepG2 and in undifferentiated PC12 cells significantly differ from each other. The mitochondrial membrane potential (MMP) represents a driving force for complex V of the mitochondrial respiration chain (F_1_/F_0_-ATPase, CV) that produces ATP [32]. Thus, lower ATP levels in HepG2 cells (Figure 2(a)) may be explained by a reduced MMP in this cell line (Figure 2(b)). The complexes CII and CI&CII of the mitochondrial respiration chain in the coupled state were virtually identical in both cell lines (Figure 2(c)). However, HepG2 cells showed significantly increased activities of complexes CIV, CII, and CI&CII if uncoupled from the MMP using FCCP. This might be due to the significantly higher mitochondrial mass of HepG2 cells, as indicated by the increased citrate synthase activity (Figure 2(d)). Citrate synthase (CS) is an enzyme of the Krebs cycle that is located in the mitochondrial matrix. It represents a robust mitochondrial mass marker [33].

### 3.2. Silibinin's Effect on Adenosine Triphosphate Levels

**Figure 3:**
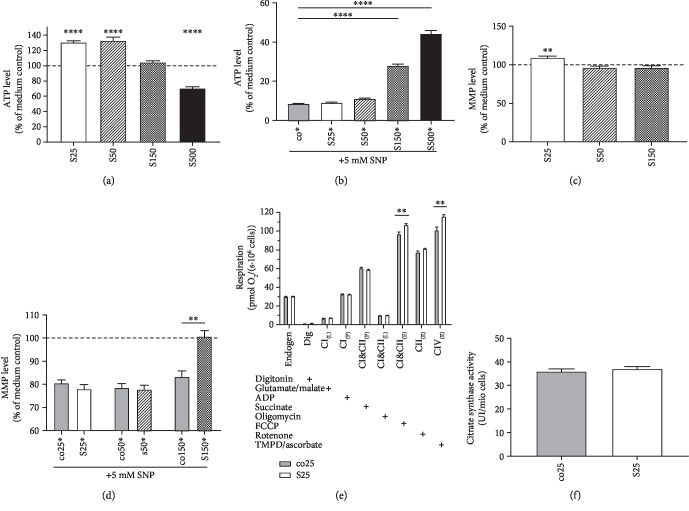
Effects of silibinin A (SIL A) on mitochondrial function in HepG2. (a) ATP level (% of medium control). Cells were incubated with SIL A (S) at different concentrations (25–150 *μ*M) for 24 h and measured against control cells treated with DMSO (data not shown). DMSO results showed no significant difference compared to the medium control. (b) ATP level (% of medium control) of cells injured via the addition of 5 mM SNP 1 hour after the initial incubation with test substances. Cells were incubated for a total of 24 h. (c) MMP level (% of medium control). Cells were incubated with 50 or 150 *μ*M SIL A for 24 h and measured against control cells treated with DMSO (data not shown). DMSO results showed no significant difference compared to the medium control. (d) MMP level (% of medium control) of cells injured via the addition of 5 mM SNP 1 hour after the initial incubation with test substances. Cells were incubated for a total of 24 h. (e) Respiration of HepG2 cells after 24 hours of incubation with the test substance or control. Measured data indicate the oxygen consumption of cells in an Oxygraph-2k (Oroboros). Cells were incubated with 25 *μ*M SIL A (S) and compared against control cells treated with DMSO (co). Activity of OXPHOS complexes was assessed via the addition of several substrates, inhibitors, or uncouplers. Which substance was added in which stage of the experiment is indicated by the placement of “+.” Dig = the addition of digitonin; CI_(L)_ = leak respiration of complex I; CI_(P)_ = coupled respiration using only complex I substrates; CI&CII_(P)_ = physiological respiration; CI&CII_(L)_ = leak respiration of complexes I and II; CI&CII_(E)_ = uncoupled respiration using maximum CI&CII activity; CII_(E)_ = uncoupled respiration using complex II substrates only; CIV_(E)_ = uncoupled respiration using only CIV after complex III inhibition and CIV activation using an electron donor. (f) Citrate synthases activity.Data are displayed as the means ± SEM. *N* = 7–18. Statistical significance was tested via one-way ANOVA and Tukey's post hoc test in (b). In (a, c–f), statistical significance was tested via Student's *t*-test of the treatment group versus the DMSO control (^∗∗∗∗^*p* < 0.0001, ^∗∗∗^*p* < 0.001, ^∗∗^*p* < 0.01, and ^∗^*p* < 0.5).

Incubation of HepG2 cells with 25 *μ*M and 50 *μ*M SIL A for 24 hours showed a significant improvement on the basal ATP level (Figure 3(a)) (*p* < 0.0001). Incubation with 150 *μ*M did not show an effect on ATP levels, and 500 *μ*M significantly reduced ATP levels, indicating a toxic effect (*p* < 0.0001). To induce nitrosative stress, cells were incubated with 5 mM SNP 1 hour after incubation with SIL A. A concentration of 150 *μ*M SIL A protected HepG2 cells from the SNP-induced drop in ATP levels (Figure 3(b)) (*p* < 0.0001). Lower concentrations, although beneficial to the basal ATP level, had no protective effect.

**Figure 4:**
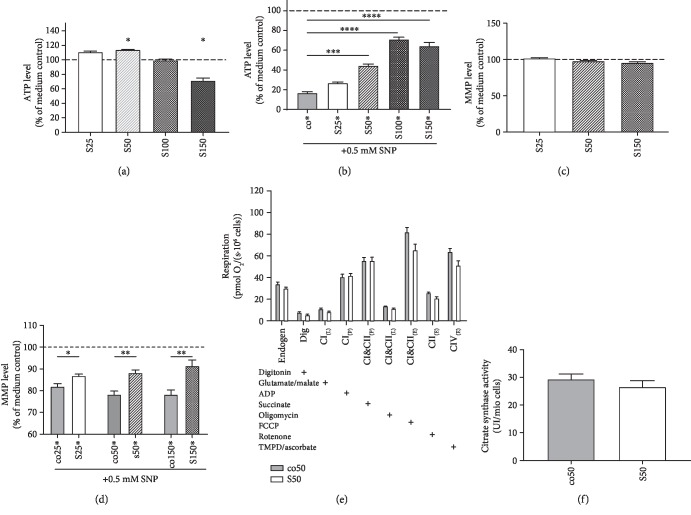
Effects of silibinin A (SIL A) on mitochondrial function in PC12 cells. (a) ATP level (% of medium control). Cells were incubated with SIL A (S) at different concentrations (25–150 *μ*M) for 24 h and measured against control cells treated with DMSO (data not shown). DMSO results showed no significant difference compared to the medium control. (b) ATP level (% of medium control) of cells injured via the addition of 0.5 mM SNP 1 hour after the initial incubation with test substances. Cells were incubated for a total of 24 h. (c) MMP level (% of medium control). Cells were incubated with 25 or 150 *μ*M SIL A for 24 h and measured against control cells treated with DMSO (data not shown). DMSO results showed no significant difference compared to the medium control. (d) MMP level (% of medium control) of cells injured via the addition of 0.5 mM SNP 1 hour after the initial incubation with test substances. Cells were incubated for a total of 24 h. (e) Respiration of PC12 cells after 24 hours of incubation with the test substance or control. Measured data show the oxygen consumption of cells in an Oxygraph-2k (Oroboros). Cells were incubated with 50 *μ*M SIL A (S) and compared with control cells treated with DMSO (co). The activity of OXPHOS complexes was assessed via the addition of several substrates, inhibitors, or uncouplers. Which substance was added in which stage of the experiment is indicated by the placement of “+.” Dig = the addition of digitonin; CI_(L)_ = leak respiration of complex I; CI_(P)_ = coupled respiration using only complex I substrates; CI&CII_(P)_ = physiological respiration; CI&CII_(L)_ = leak respiration of complexes I and II; CI&CII_(E)_ = uncoupled respiration using maximum CI&CII activity; CII_(E)_ = uncoupled respiration using complex II substrates only; CIV_(E)_ = uncoupled respiration using only CIV after complex III inhibition and CIV activation using an electron donor. (f) Citrate synthases activity. Data are displayed as the means ± SEM. *N* = 6–12. Statistical significance was tested via one-way ANOVA and Tukey's post hoc test in (b). In (a, c–f), statistical significance was tested via Student's *t*-test of the treatment group versus the DMSO control (^∗∗∗∗^*p* < 0.0001, ^∗∗∗^*p* < 0.001, ^∗∗^*p* < 0.01, and ^∗^*p* < 0.5).

In PC12 cells, SIL A seemed to have little to no effect on basal ATP levels. Incubation of PC12 cells with 25 *μ*M and 100 *μ*M SIL A for 24 h showed no difference in ATP levels. However, cells treated with 50 *μ*M SIL A showed a significant increase in ATP (*p* = 0.0411). Compared to HepG2 cells, PC12 cells seemed to be more vulnerable to SIL A, since incubation with 150 *μ*M SIL A significantly reduced basal ATP levels (*p* < 0.0298). PC12 cells were also more vulnerable to nitrosative stress. When cells were incubated with 0.5 mM SNP, this significantly reduced ATP levels to around 20% (Figure 4(b)), similar to the result observed for 5 mM SNP in HepG2 cells. Incubation of cells with 25 *μ*M, 50 *μ*M (*p* = 0.0003), 100 *μ*M (*p* < 0.0001), and 150 *μ*M (*p* < 0.0001) SIL A 1 hour prior to SNP showed a concentration-dependent, protective effect in PC12 cells after 24 h (Figure 4(b)).

### 3.3. Mitochondrial Membrane Potential

Except for the concentration of 25 *μ*M SIL A (*p* = 0.0042), basal MMP levels of HepG2 cells were unaffected by SIL A treatment (Figure 3(c)). Nitrosative stress was induced by incubation with 5 mM SNP 1 hour after incubation with SIL A. If cells were treated with 150 *μ*M SIL A, the effect of SNP-induced stress could be significantly attenuated (*p* < 0.0018) (Figure 3(d)).

Incubation of PC12 cells with 25 *μ*M, 50 *μ*M, or 150 *μ*M SIL A had no effect on the basal MMP. However, as can be seen in Figure 4(d), SIL A significantly reduced the damage caused by SNP-induced nitrosative stress (25 *μ*M *p* < 0.0487, 50 *μ*M *p* < 0.0021, and 150 *μ*M *p* < 0.0057).

### 3.4. Respiration

Incubation of HepG2 cells with 25 *μ*M SIL A for 24 hours significantly enhanced the respiration of complexes CI&CII (in the uncoupled state) (*p* = 0.0093) and CIV (in the uncoupled state) (*p* < 0.0045) of the mitochondrial respiration chain (Figure 3(e)). In PC12 cells, SIL A had no effect on the oxygen consumption of the complexes in the oxidative phosphorylation system (Figure 4(e)).

### 3.5. Citrate Synthase Activity

Incubation with SIL A had no effect on HepG2 cells (Figure 3(f)) nor on PC12 cells (Figure 4(f)).

### 3.6. Effect of Silibinin on Glycolysis

Besides mitochondrial respiration, cells also produce ATP by glycolysis. Enhanced glycolysis might explain the enhanced ATP levels in PC12 cells after SIL A incubation, although respiration was not changed (Figures 4(a) and 4(e)).

As seen in Table 1, neither pyruvate nor lactate levels were significantly affected by SIL A treatment in PC12 cells. However, in HepG2 cells, SIL A significantly reduced lactate concentrations (*p* = 0.0007) and a reducing trend of pyruvate levels was also shown. The ratio of pyruvate/lactate was not affected in either cell line.

## 4. Discussion

In hepatic HepG2 cells, SIL A enhanced the activity of respiratory complexes and subsequently may be responsible for an increase in the production of ATP. In PC12 cells, SIL A had no effect on respiration. However, treatment with SIL A tended to decrease the respiration of CI&CII_(E)_ and CIV_(E)_ activity, whereas CI&CII_(P)_ activity was virtually identical in both groups. These findings suggest that SIL A did not affect the activity of both complexes. In HepG2 cells, on the other hand, we found a significant increase in CI&CII_(E)_ and CIV_(E)_ activities. It has to be noted, however, that the uncoupling of the respiration chain from the MMP, indicating the maximum possible oxygen consumption, is an artificial state, which does not occur under normal cell conditions [34]. In an abundance of intramitochondrial ADP, CI&CII_(P)_ coupled respiration better reflects physiological respiration. This state, however, was unaffected by SIL A in both cell lines. Additionally, our results for citrate synthase activity as a marker for mitochondrial content [33, 35] showed that the increased ATP concentrations cannot be linked to increased mitochondrial mass.

Although there was no significance or trend for increased oxidative phosphorylation (OXPHOS) activity, we also did not find any indication of an impaired respiratory system. Since there have been multiple studies [36–38] showing an increased production of ROS following SIL A treatment and an impairment of respiration at high concentrations of ROS [25], our data showed that SIL A did not affect mitochondrial respiration in low concentrations. SIL A also had no influence on pyruvate or lactate concentrations, indicating that the glycolytic pathway was not affected by SIL A treatment. This is in agreement with the lack of inhibition of respiration in PC12 cells, as there is no compensatory activation of the glycolytic pathway, as found by Liemburg-Apers et al. in myoblasts with restricted respiration [39]. In HepG2 cells, we found a significant reduction in lactate levels, although pyruvate remained unchanged. This may indicate a compensatory downregulation of glycolysis induced by increased ATP levels. Our data showed that increased cellular ATP concentrations were not linked to the respiratory system. Although the underlying mechanisms are not yet identified, SIL A may affect different cellular processes linked to ATP use, as it may be mainly produced by the mitochondria, though used throughout the cell for a host of different bioenergetic processes.

As citrate synthase activity was not affected by SIL A treatment, mitochondrial biogenesis in PC12 and HepG2 cells might also not be altered by SIL A.

Table 2 shows that SNP-induced nitrosative stress caused severe damage to the mitochondria of hepatic and neuronal cells and significantly reduced MMP. Since it has been shown that higher concentrations of RNS can inhibit not only complex IV of the respiratory chain but also complexes I and III [26, 40], these results were expected. Further, these changes led to a decreased production of ATP at complex V. We used hepatic HepG2 cells due to their low expression of Cyp450 enzymes and thus their low metabolism of xenobiotics [41] to gain better insight into the effect of SIL A on the cells. However, in order to achieve similar damage, SNP concentrations differed tenfold between the two cell lines. Since the liver is characterized by a complex system eliminating ROS/RNS [42] and has increased resistance to toxins [43], it is difficult to compare both cell lines due to their metabolic differences. This is also reflected in the differences in mitochondrial parameters between the two cell lines shown in Figure 2. For example, we found a significant difference in mitochondrial mass, which was expected since hepatic mitochondria play an important role in the beta oxidation of lipids. As reported by Liu et al., we also found a strong protective effect of SIL A against nitrosative stress on both cell lines [15].

In PC12 cells, the attenuation of nitrosative stress already tended to appear at low concentrations of 25 *μ*M, while in hepatic cells, no effect could be found below 150 *μ*M. These significant effects could be explained by findings that silibinin is a potent scavenger for a host of free radicals *in situ* [44]. Furthermore, SIL A has been found to increase superoxide dismutases (SOD) and glutathione peroxidase (GPx) activity in human erythrocytes [45]. Liu et al. found that treatment with SIL A generated increased levels of ROS, activating antiapoptotic pathways and therefore protecting against SNP-induced damages [15]. Since ROS also plays a key role in the balance of free radicals and scavengers, potentially increasing oxidative stress, concentrations that are too high lead to toxic effects. Our data are in support of this assumption, as we observed toxic effects on basal ATP levels at a concentration of 150 *μ*M in PC12 cells and a concentration of 500 *μ*M in HepG2 cells. This agrees with the results of Matsuo et al., who also found toxic levels of ROS due to the application of high flavonoid concentrations in human cells [46, 47]. It should be noted, however, that although 500 *μ*M SIL A had a toxic effect on basal ATP, the protective effects against SNP-induced damage still increased compared to 150 *μ*M SIL A.

Lower concentrations of 25 *μ*M and 50 *μ*M SIL A attenuated nitrosative damages as well as increased basal levels of ATP. These concentrations are similar to the unconjugated SIL A concentrations found in the plasma of rats (18 *μ*M) after the oral administration of 500 mg/kg SIL A [48]. In humans, however, SIL A is best administered as silibinin-phosphatidylcholine complex, resulting in a mean plasma concentration of 75 *μ*M in human cancer patients [49].

In conclusion, we report that effective concentrations of SIL A attenuated nitrosative stress in both PC12 and HepG2 cells. Furthermore, low concentrations of SIL A improved basal MMP and ATP levels in HepG2 cells, though less so in PC12 cells. SIL A enhanced uncoupled mitochondrial respiration in HepG2 cells but did not affect the mitochondrial content of either cell line. Based on our findings that SIL A protects against SNP-induced nitrosative stress at the level of mitochondria, we conclude that SIL A might be beneficial against neurological disorders although it did not provide a general improvement of the mitochondrial function.

## Figures and Tables

**Table 1:** Pyruvate and lactate concentrations adjusted to the protein content of samples of either PC12 or HepG2 cells. Data are displayed as the means ± SEM. *N* = 9. Significance was tested via Student's *t*-test.

	Pyruvate/protein content (*μ*mol/mg)	Lactate/protein content (*μ*mol/mg)	Pyruvate/lactate ratio
PC12			
Control	0.4962 ± 0.05464	5.314 ± 0.8064	11.33 ± 0.6850
SIL A 50 *μ*M	0.4883 ± 0.02037	5.056 ± 0.4885	10.91 ± 1.004
Significance	*p* = 0.8942	*p* = 0.7888	*p* = 0.7420
HepG2			
Control	0.5124 ± 0.04212	5.388 ± 0.1372	10.34 ± 0.7984
SIL A 50 *μ*M	0.4487 ± 0.05332	4.515 ± 0.1336	10.92 ± 1.083
Significance	*p* = 0.3677	^∗∗∗^ *p* = 0.0007	*p* = 0.6728

**Table 2:** Summary of the effects of silibinin on mitochondrial parameters in HepG2 and PC12 cells.

	Respiration	Basal MMP/+SNP	Basal ATP/+SNP	Citrate synthase activity	Glycolysis
HepG2	↑	↑/↑	↑↑/↑	↔	↓
PC12	↔	↔/↑↑	↑/↑↑	↔	↔

↑ indicates an increase, ↓ indicates a reduction, and ↔ indicates no change. SNP = sodium nitroprusside.

## Data Availability

The dataset generated during this study is available from the corresponding author upon reasonable request.
